# A Systematic Review of Depression and Anxiety in Patients with Atrial Fibrillation: The Mind-Heart Link

**DOI:** 10.1155/2013/159850

**Published:** 2013-04-27

**Authors:** Dimpi Patel, Nathaniel D. Mc Conkey, Ryann Sohaney, Ashley Mc Neil, Andy Jedrzejczyk, Luciana Armaganijan

**Affiliations:** ^1^Mercy Street Vincent's Medical Center, 2213 Cherry St., Toledo, OH 43608, USA; ^2^Electrophysiology and Cardiac Arrhythmias, Dante Pazzanese Institute of Cardiology, 04012-909 SP, Brazil

## Abstract

Atrial fibrillation (AF) is the most commonly seen arrhythmia in clinical practice. At present, few studies have been conducted centering on depression and anxiety in AF patients. Our aim in this systematic review is to use the relevant literature to (1) describe the prevalence of depression and anxiety in AF patients, (2) assess the impact that depression and anxiety have on illness perception in patients with AF, (3) provide evidence to support a hypothetical connection between the pathophysiology of AF and depression and anxiety, (4) evaluate the benefit of treatment of AF on depression and anxiety, and (5) give insight on medically managing a patient with AF and concomitant depression and anxiety.

## 1. Introduction

Atrial fibrillation (AF) is a cardiovascular epidemic which affects more than 3 million individuals in the United States alone [1, 2]. Diabetes, congestive heart failure, hypertension, aging of the population, male gender, and obesity are just a few risk factors which increase the incidence of AF [3, 4]. AF is associated with significant morbidity and mortality. 

At present, many conducted studies assess the impact of AF on health-related quality of life (HRQOL) [5]. However, limited information on depression and anxiety in patients with AF exits. 

Our aim in this comprehensive systematic review is to use relevant literature to the (1) describe the prevalence of depression and anxiety in AF patients, (2) assess the impact that depression and anxiety have on illness perception in patients with AF and vice versa, (3) provide evidence to support a hypothetical connection between the pathophysiology of AF and depression and anxiety, (4) evaluate benefit of treatment of AF on depression and anxiety, and (5) give insight on medically managing a patient with AF and concomitant depression and anxiety. 

## 2. Methods

### 2.1. Data Sources and Searches

A detailed literature search was conducted using electronic databases including PubMed, MD Consult, and PsycINFO from their inception through January 2012. One of the investigators, with the help of a qualified medical librarian, did the electronic search. All designs of studies (observational, cross-sectional, case-control, and cohort studies) on depression and anxiety in AF patients were considered. The following search key terms were used: atrial fibrillation, depression, anxiety, catheter ablation, cardioversion, and antiarrhythmic drugs. Only articles printed in the English language were included. Once studies were retrieved, abstracts were screened, followed by full-article review and assessment for inclusion. We also manually searched the reference lists of the included studies to ensure a comprehensive search of the literature. Studies were appraised and selected by two reviewers (D. Patel, A. Jedrzejczyk). Disagreements on the inclusion/exclusion of the study were solved by consultation with a third reviewer (R. Sohaney).

### 2.2. Study Selection

Prospective and retrospective studies were included. We excluded reviews, editorials, letters, case series, case reports, and conference proceedings. We only included studies which (1) clearly defined an AF study population, (2) had at least 1 of the study variables as depression or anxiety, (3) used a tested instrument which specifically measured depression or anxiety, (4) identified clear outcomes, (5) had no selective loss during the followup, and (6) identified important confounders. 

## 3. Results

The literature search identified 565 citations. After reviewing the titles and the abstracts of the 565 citations, we found 34 articles that focused on AF and depression and anxiety. Seven articles were removed due to (1) foreign language, (2) review articles, (3) letters, and (4) failure to use a validated instrument to measure depression and anxiety [6–12]. 

**Figure 1:**
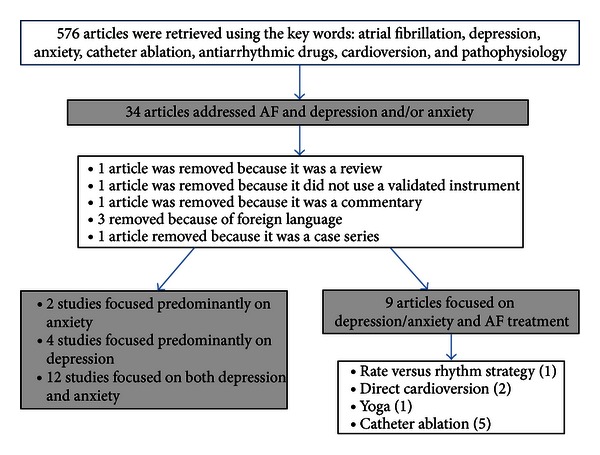
Schematic of the literature search for articles on depression and/or anxiety and atrial fibrillation.

Of the articles that did not focus on AF treatment, 4 studies only used instruments to measure depression [13–16], 2 studies assessed anxiety [17, 18], and 12 assessed both depression and anxiety [19–30]. There were 8 articles that addressed AF treatment strategies and depression and anxiety in some context. Six were catheter ablation studies, 2 on rate and/or rhythm strategies, and 1 on yoga [31–38] (Figure 1).

### 3.1. Epidemiology of Depression and Anxiety in AF Patients

Most studies assessing depression and anxiety in cardiac patients have been conducted in a coronary artery heart disease population [39–41]. However, patients with AF in particular have been shown to suffer from an increased prevalence of psychological distress. A study by Thrall et al. found that 38% of subjects with AF met criteria for significant depression under the Beck Depression Inventory (BDI). Furthermore, 28% and 38% were considered to possess state and trait anxiety in accordance with the State-Trait Anxiety Inventory (STAI). While their level of depression was not significantly higher than that of patients of another chronic disease examined in the study (hypertension), trait anxiety was markedly greater (38% for AF patients versus 22% for hypertensive patients, *P* = 0.03). Depression and anxiety levels correlated with the quality of life in patients with AF. Female and unemployed patients with AF had significantly poorer quality of life. Moreover, levels of both depression and anxiety in patients with AF did not show significant change at 6-month followup [19]. 

While heightened levels of depression are not unique to AF patients, it is significant nonetheless. Using the BDI, Dabrowski et al. also found that patients with AF have significantly higher rates of depression than members of the general population. While their control group experienced depression with a prevalence of 5.7%  ± 5.8%, patients with paroxysmal, persistent, and permanent AF showed depression in 10.8%  ± 5.8%, 10.0%  ± 6.4%, and 10.1%  ± 7.2%, respectively. Patients with AF reported significant declines in satisfaction related to work, sex life, household activities, social life, and leisure time due to disease-related limitations [13]. 

Dabrowski et al. also reported that women with AF significantly suffered from more depression, sleep problems, and physical manifestations than males [13]. Ong et al. also found that females with AF have lower physical quality of life relative to male patients, and this relationship may be mediated by self-reported symptoms of depression [20]. 

Frasure-Smith et al. reported in a substudy from the AF-CHF trial of rate—versus rhythm control strategies that elevated depression symptoms predict long-term cardiovascular mortality in patients with AF and heart failure. Depression symptoms were assessed using the BDI-II. Thirty-two percent of patients had elevated depression scores. Cox proportional hazard models adjusted for prognostic factors such as age, marital status, cause of CHF, and previous AF hospitalization, among others showed that elevated depression scores significantly predicted cardiovascular mortality, arrhythmic death, and all-cause mortality in patients with AF and CHF. Interestingly, unmarried risk associated with depression and marital status were additive with depressed unmarried patients at the greatest risk for mortality [14].

 Perret-Guillaume et al. further concluded that the psychological impact of AF may have even more clinical consequence than its physical manifestations. In defense of such conclusions, they present data collected from elderly populations via the Duke Health Profile that showed significant differences in mental function, depression levels, and anxiety in AF patients relative to controls. Conversely, the same instrument found no statistically significant differences in measures of physical health, social impairment, or disability [21]. 

On the other hand, a similar study by Ariansen et al. assessing an elderly population (>75 years, *n* = 27) failed to find significantly higher prevalence of depression and anxiety in AF patients using the Health, Anxiety, and Depression Scale (HADS). The authors concluded that physical implications account for the diminished quality of life in AF patients using the SF-36. Perhaps the difference in findings could be attributed to the fact that Ariansen et al. chose to include only patients with permanent and clinically stable AF [22] (Table 1). 

### 3.2. Illness Perception and Resultant Depression and Anxiety and Vice Versa

The extent of psychological and/or physical distress in AF patients appears to be correlated to how patients perceive their illness, personality traits and effect, illness management style, and degree of somatic preoccupation. Trovato et al. used an Illness Perception Questionnaire (IPQ-R) and Personal stress levels (per PSM) and found that stress was higher in patients with less favorable perceptions of their condition. In particular, perceptions regarding the timeline of the disease, its perceived impact on their anxiety level, and the efficacy of treatment correlated with stress levels. Women had higher PSM and HADS anxiety scores. However, they had similar HADS depression scores as men. Of note, level of education, ability to identify symptoms, and degree of personal control did not significantly predict stress levels. Interestingly, coffee consumption was associated with lower stress levels in patients with AF [23]. 

In a larger study by McCabe et al., 207 patients were given the IPQ-R and assessed for subjective feelings of worry, anxiety, and depression. It was found that the perceptions most strongly associated with negative emotional status were that AF was cyclic and unpredictable, was caused by psychological factors such as stress or worry, and had great clinical consequence. Conversely, positive emotional status was predicted by perceptions of AF as well-understood, controllable with treatment, and of less clinical consequence. The author suggests that efforts to ascertain the patient's degree of understanding and perceptions of his or her illness should be made in order to identify patients likely to require management of depression or anxiety [24]. 

Ong et al. found that personality traits and illness management styles were important factors in quality of life in AF patients. Optimism was correlated with better quality of life and lower distress; however, it was unrelated to physical quality of life and symptom severity [25]. Interestingly, Whang et al. found that positive effect was associated with a lower risk of AF [26]. Ong et al. also reported in patients with high anxiety sensitivity, AF symptoms can possibly create a maladaptive cycle of hypervigilance and somatic preoccupation which results in disengagement from daily activity and subsequently poorer quality of life [25]. 

In a study by Lane et al., 70 patients were assessed for depression and anxiety change over the first 12 months following diagnosis of lone AF. They additionally investigated whether illness perceptions and beliefs about medication at the time of diagnosis are associated with health related quality of life and effective response over time. They found that patients with lone AF reported few depressive and more anxiety symptoms. Patients who perceived more stress at the time of diagnosis had better improvement in mental quality of life and state anxiety symptoms over time. This could be attributed to the possibility that they sought medical advice for their anxiety. The more symptoms that a patient attributed to their AF at baseline related to a poorer improvement in physical health over time. The more concerned the patient was about medications and its effects, the lesser decline they had in physical health over time [27].

Kang used the Center for Epidemiological Studies of Depression Scale (CES-D) to measure depression and the Mishel Uncertainty in Illness Scale—Community Form (MUIS-C) to measure uncertainty in AF patients. The latter instrument is unique in that it draws attention to the fact that, in the context of disease, “uncertainty” can yield one of two conclusions in a patient who appraises it: uncertainty may be perceived as either a danger or an opportunity. Put another way, if one patient interprets their chest pain as a heart attack while another considers it indigestion, the presence of uncertainty in their self-diagnosis will be reassuring for the former but worrisome for the latter. A statistically significant correlation was thus found between the level of uncertainty as a *danger* and depression, while a negative statistically significant correlation was found in uncertainty as an *opportunity* and depression. However, the author makes it clear that, among those who experience some degree of uncertainty in the context of their atrial fibrillation, they are far more likely to perceive it as a danger [15].

**Figure 2:**
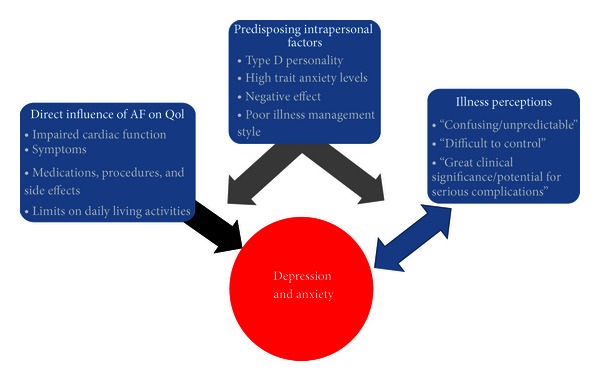
Patients with AF have impaired cardiac function, are symptomatic, have to take medications or undergo procedures, and have limits on daily living activities resulting in depression and anxiety and subsequent poorer quality of life. Additionally, predisposing intrapersonal factors such as negative effect (pessimism) or poor illness management style can further contribute to depression or anxiety. Perceiving AF illness as confusing, unpredictable and having the potential for complications can result in higher levels of depression and anxiety in patients. Moreover, depression and anxiety impact illness perception.

 Gehi et al. found that patients were more likely to score higher on the Toronto Atrial Fibrillation Severity Scale (AFSS) in the presence of either depression or anxiety irrespective of arrhythmia burden. The authors concluded that poorer AFSS scores are likely the result of patients attributing a disproportionately high amount of their distress to their organic illness, not necessarily an actual progression in the severity of their atrial fibrillation. Regardless, these patients consumed more healthcare resources in pursuing treatment targeted at their disease than those without comorbid psychiatric illness. Specifically, a correlation was found between depression and “AF Visit Score (AFVS),” a measure of the frequency of visits to physician offices and emergency rooms illustrating the vicious cycle by which certain patients transfer illness-related psychological distress back into their perception of bodily dysfunction. The study also identified a tendency for patients with atrial fibrillation to suffer from somatization disorder as well [28] (Figure 2 and Table 2). 

### 3.3. The Connection between the Pathophysiology of AF and Depression and Anxiety

Most studies have shown that patients with AF have an increased incidence of depression and anxiety due to impairment in quality of life. However, whether depression and anxiety trigger AF has not been thoroughly investigated. At present, we know from Eaker et al. that anxiety is a risk factor for incident AF in males and females over a 10-year time period [18]. On the other hand, Whang et al. report that depression is not a risk factor for incident AF in women. However, the study by Whang et al. did not use detailed instruments to measure depression, incidence of AF was reported by the patient and then validated by medical record so episodes could be missed, and biomarkers for AF were not collected [26]. Therefore, there is too limited information to comment conclusively on whether depression and anxiety can trigger AF. 

Nonetheless, it has been hypothesized that inflammation and oxidative stress are culprits in the initiation and perpetuation of AF [42]. Depressed patients have increased levels of acute phase reactants, such as C-reactive proteins, proinflammatory cytokines, and decreased levels of anti-inflammatory molecules [43]. Rommel et al. reported that in unadjusted analyses, mild-to-moderate and severe depression were associated with increased hs-CRP compared to no or minimal depression in an AF population. However, on multivariate analysis, depression was no longer associated with increased hs-CRP (*P* value = 0.187 in mild depression; *P* value = 0.094 in moderate depression) [16]. Son and Song reported that AF patients with a “Type D” personality are more likely to have elevated levels of hs-CRP relative to patients of non-type-D personality [29]. Prior studies have shown that higher CRP levels are associated with incidence or recurrence of AF [44–46]. Importantly, the studies by Son and Song and Tully et al. do not prospectively establish that depression triggers new onset AF since these studies have been conducted in patients who already have a diagnosis of AF [29, 30]. 

Additionally, patients who suffer from depression and anxiety have increased activation of the sympathetic nervous system [47]. Hansson et al. report that psychic stress which causes catecholamine release was a common inciting factor in patients hospitalized with paroxysmal AF. However, this study did not use a validated psychologic instrument to assess for anxiety, and it was based on patient beliefs of what triggered the AF episode [10]. Tully et al. reported that anxiety symptoms increased the incidence of AF after cardiac surgery. Increased sympathetic tone and decreased vagal tone, which can be caused by anxiety, have been noted prior to postoperative AF, before the onset of atrial flutter and the onset of lone AF [30]. 

**Figure 3:**
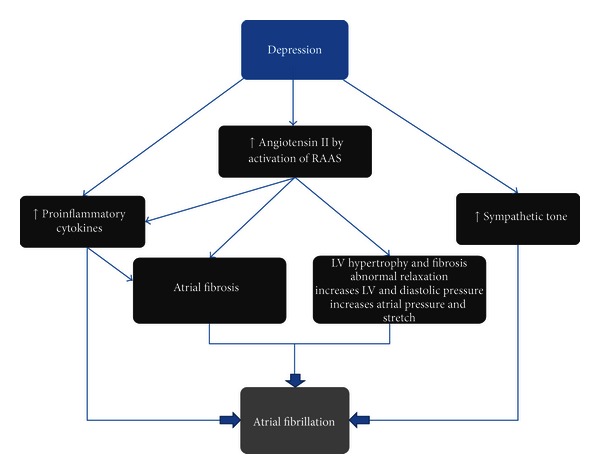
A hypothetical model of how depression can initiate atrial fibrillation.

Moreover, patients who suffer from depression have activation of the hypothalamic-pituitary-adrenal axis and the renin-angiotensin-aldosterone system [48]. Elevated levels of angiotensin II stimulate mitogen activated protein kinases and reduction in collagenase activity which result in cardiac fibrosis formation. Additionally, angiotensin II binds to angiotensin II type I receptors which stimulate transforming growth factor (TGF)-*β*1 production which promotes atrial fibrosis. A dilated left atrium promotes AF by slowing atrial conduction velocity and providing a greater area for reentry. Angiotensin II induces the production of reactive oxygen species, inflammatory cytokines, and adhesion molecules. ACE inhibitors reduce C-reactive protein, TNF-*α*, and IL-6 in hypertensive patients [49]. In short, while there is not enough evidence to clearly say that depression and anxiety trigger new onset AF, it is tempting to infer that these comorbidities create a milieu that is conducive to the initiation and perpetuation of AF (Figure 3 and Table 3). 

### 3.4. The Benefit of AF Treatment on Depression and Anxiety and Impact of Depression and Anxiety on Treatment Success

AF treatment strategies include rate and rhythm pharmacologic agents, electrical cardioversion, and catheter ablation. While it is still debated, rate and rhythm control have been shown to be equal in terms of mortality benefit. Moreover, studies which compare quality of life between the two treatment approaches have been similar [50, 51]. The AF-CHF trial of rate versus rhythm control reported that there was no rhythm versus rate control benefit in the prevention of arrhythmic death in patients with elevated depression symptoms [14]. In another study, Frasure-Smith et al. reported in patients with CHF and AF and high anxiety sensitivity scores who were assigned to a rhythm-control group significantly lower cardiovascular mortality than those receiving rate control [31].

Restoration of sinus rhythm via electrical cardioversion is associated with improvement in quality of life [52]. However, like pharmacologic rate and rhythm strategies, the effect that electrical cardioversion has on depression and anxiety symptoms in AF patients has yet to be investigated. Nonetheless, Lange and Herrmann-Lingen reported that depressive symptoms are a major risk factor for recurrence of AF after successful electrical cardioversion. Patients who scored higher than 7 on the HADS had 85% recurrence of AF compared to 39% of nondepressed patients. HADS anxiety scores and the presence of type D personality were not associated with recurrence of AF. Lange and Herrmann-Lingen postulated that heightened adrenergic tone and proinflammatory state may be responsible for the increased recurrence rates, further giving credence to the possibility that depression may trigger AF [32]. 

Catheter ablation is safe and efficacious for AF and is being increasingly performed. Fichtner et al. studied patients who had undergone catheter ablation assessing long- and short-term benefit. They used 7 different validated generic and specific tools to quantify change in quality of life. During short- and long-term followup all patients with paroxysmal or persistent AF showed a significant quality of life improvement in all 7 tools, irrespective of catheter ablation success [33]. Similarly, Wokhlu et al. reported that quality of life improvement was not solely associated with ablation efficacy and factors such as baseline quality of life, discontinuation of anticoagulation drugs, and symptom relief played a role as well [34]. Additionally, Fichtner et al. reported that in long-term follow-up, patients with successful ablation had more improvement in disease-specific questionnaires such as AF severity scale, AF symptom checklist, and in the major depression inventory compared to patients with unsuccessful ablation [33]. This improvement in quality of life in both studies could be due to placebo effect for short-term results; however, this would not apply to long-term results. Most likely, patients felt better because they were in sinus rhythm or had a reduction in AF burden and were free of medications. 

Sang et al. assessed improvement in depression, anxiety, and quality of life in patients who underwent catheter ablation compared to those treated with antiarrhythmic drugs. They reported that catheter ablation was effective in reducing symptoms of depression and anxiety and improving quality of life, and it was superior to antiarrhythmic drug therapy [35].

Mohanty et al. and Yu et al. both assessed improvement in depression and anxiety in patients undergoing catheter ablation [36, 37]. Mohanty et al. reported that successful ablation had greater improvement in the Hospital Anxiety and depression scale and BDI scores, whereas the STAI scores did not show any association with ablation success [36]. Yu et al. also noted that depression and anxiety increases the recurrence risk of persistent AF after circumferential pulmonary vein ablation [37]. 

Very little information currently exists on the efficacy of alternative therapies in improving anxiety and depression in AF. Recently, Lakkireddy et al. reported that yoga improves anxiety and depression in patients with paroxysmal AF. Doing yoga significantly reduces the number of symptomatic and asymptomatic AF episodes. Yoga may prevent the initiation of AF through pleiotropic effects such as increasing baseline parasympathetic tone, suppressing extreme fluctuations in the autonomic nervous system, and reduction in atrial remodeling. In patients with paroxysmal AF, yoga training can be considered as a low-cost complement to conventional therapy in the treatment of anxiety, depression, and the symptomatic burden of AF [38] (Table 4).

## 4. Conclusion

There is a complex relationship between depression, anxiety, and AF. AF can cause depression and anxiety in patients, and depression and anxiety may create an environment that is conducive for the initiation and perpetuation of AF. Importantly, depression and anxiety affect how patients perceive their illness, particularly for women, and impact healthcare utilization. The presence of depression and anxiety can impact the effectiveness of certain AF treatments. Therefore, implementing strategies which can reduce anxiety and depression in our AF patients may improve treatment outcomes, patient quality of life, and reduce financial burdens associated with AF. Such strategies include patient education of the disease process thereby reducing uncertainty, management of AF symptoms aggressively, catheter ablation when antiarrhythmic drugs fail, and perhaps treating patients with psychiatric medications. 

Using antidepressants in AF patients to control depression and anxiety symptoms or to prevent AF has not been well studied. However, there was a very small study which found that paroxetine reduces drug resistant paroxysmal AF. The authors suggested that paroxetine can modulate vagal tone at the level of the midbrain and inhibit the vasovagal reflex thus terminating AF [53]. On the other hand, there have been a few isolated reports of new onset AF with SSRI use [54, 55]. 

Treating depression and anxiety in AF patients have certain challenges. For example, amiodarone routinely prolongs QT intervals and as does citalopram and escitalopram. A single case reported an adverse interaction of amiodarone and citalopram which resulted in torsades de point [56]. However, the incidence of citalopram induced torsades de pointe is small and most SSRIs do not cause QT prolongation so can safely be used with amiodarone [57]. Nonetheless, clinicians should be cautious in prescribing certain SSRIs and amiodarone in elderly females, patients with advanced cardiac disease, or those on diuretics that cause hypokalemia because they are prone to increased risk of QT prolongation [58]. This is particularly clinically relevant in the context of an AF population because females typically present with AF later in life and have higher prevalence of depression which may require treatment with SSRIs. Additionally, patients with AF and CHF often also suffer from depression, and many of them are already treated with a diuretics and amiodarone. 

Another challenging aspect of treating AF patients with SSRI centers on anticoagulation issues. Warfarin interacts with paroxetine, venlafaxine, fluoxetine, and duloxetine thus increasing PT [59]. Some SSRIs are associated with increased bleeding risk [60, 61]. When SSRIs are used in combination with warfarin the risk of any bleed, major bleeding, and hospitalization secondary to bleeding is increased compared to patients solely on warfarin; therefore, clinicians need to be more vigilant in this population [59]. There is also an increased risk of bleeding with dabigatran and SSRIs/NSRIs [62]. 

 At present, there are many unanswered questions on how to best clinically manage patients with AF, depression, and/or anxiety. Further trials are necessary to elucidate the benefits of SSRI use in the prevention of incident AF and quality of life in AF patients, the impact in terms of reduction of depression and anxiety levels, and if depression or anxiety directly cause new onset AF. 

## Figures and Tables

**Table 1:** A summary of studies presented in the Epidemiology depression and anxiety in AF Patients section. Studies in italics only used a depression tool, and articles in bold only assessed anxiety.

Study	Subject size	Aim of the study	Psychological test	Significant findings
Epidemiology of depression and anxiety in AF patients				

Thrall et al. [19] (2007)	101 patients with AF were compared to hypertensive patients	To report the prevalence of depression and anxiety in patients with AF	(i) Trait and State Anxiety (ii) Beck Depression Inventory	(i) 28% had state anxiety (ii) 38% had trait anxiety (iii) 38% had depression

*Dabrowski et al. [13] (2010) *	*150 patients with paroxysmal, persistent, and permanent AF *	*To report quality of life and depression level in patients with various patterns of AF *	*(i) Nottingham Health Profile questionnaire* *(ii) Beck Depression Inventory *	*(i) AF patients had a higher risk of depression * *(ii) Patients with paroxysmal and permanent AF had lower self-evaluation of their energy level * *(iii) AF limited quality of life, sexual life, and professional and home activity *

Ong et al. [20] (2006)	93 patients with AF	To report the relationship between gender, depression, AF severity	(i) Anxiety and Depression (HADS) (ii) Toronto AF Severity Scale	(i) 11% of patients suffered from depression (ii) Women had higher depression scores

*Frasure-Smith et al. [14] (2009) *	*974 patients with AF and CHF *	*To report if depression predicts long-term cardiovascular mortality in patients *	*Beck Depression * *Inventory II *	*(i) 32% had BDI-II scores ≥14 (at least mild-to-moderate symptoms of depression)* *(ii) Women, nonwhite, unmarried, and those with lower levels of education had more depression * *(iii) Depression symptoms are related to increased cardiovascular mortality *

Perret-Guillaume et al. [21] (2010)	41 patients with AF were compared to 123 control patients	To compare HRQoL in AF elderly inpatients with that of age-matched controlled subjects.	(i) MOS-SF 36 (ii) The Duke Health Profile	More patients with AF suffered from depression and anxiety

Ariansen et al. [22] (2011)	27 patients with permanent AF to 75 patients in sinus rhythm	To report if permanent AF patients have more anxiety, depression, and sleep impairment than patients in sinus rhythm	(i) Hospital Anxiety and Depression Scale (HADS) (ii) Pittsburgh Sleep Quality Index (PSQI) score. (iii) Short Form 36 (SF-36)	Elderly permanent AF patients had similar levels of anxiety, depression, and sleep quality

**Suzuki and** **Kasanuki** ** [17] (2004)**	**240 patients with paroxysmal AF**	**To report the impact of symptoms of anxiety attack and anxiety on HRQOL**	**(i) Trait Anxiety (STAI)**	**(i) Trait anxiety was a predictor of poorer HRQOL** **(ii) Anxiety caused fear of attacks and agoraphobic symptoms**

**Table 2:** A summary of the articles covered in the illness Perception and Resultant Depression and Anxiety and Vice Versa section of the paper. Studies in italics used only a depression tool.

Study	Subject size	Aim of the study	Psychological test	Significant findings
Illness perception and depression and anxiety in patients with AF				

Trovato et al. [23] (2012)	45 women and 35 men with AF were compared	To report if perceived stress in stable AF has any correlation to gender and lifestyle choices	(i) Psychological Stress Measure (PSM) test (ii) Illness Perception Questionnaire (IPQ-R) (iii) Generalized Self-Efficacy scale (GSE) (iv) Hospital Anxiety and Depression Scale (HADS)	(i) Psychological stress is greater in women in comparison with men (ii) Women showed no difference in depression HAD scores. (iii) Coffee consumption was associated with lower stress levels in patients with AF

McCabe et al. [24] (2011)	207 patients with AF	To describe illness beliefs in patients with recurrent symptomatic AF and relationships among illness beliefs having implications for self-management	Illness Perception Questionnaire (IPQ-R)	(i) Patients believed psychological factors, age, and heredity caused AF (ii) Patients reported that AF induced anxiety and depression. (iii) Patients with a good understanding of AF had fewer negative emotions

Ong et al. [25] (2006)	93 patients with AF	To report the impact of personality traits and symptom preoccupation on HRQOL and psychological distress	Anxiety and Depression (HADS)	(i) Patients who had lower levels of optimism had more symptom preoccupation and severity of symptoms. (ii) Anxiety sensitivity was related to poorer HRQOL and psychological distress

Whang et al. [26] (2012)	30, 746 women without history of cardiovascular disease or AF	To assess psychological distress and risk of AF in the Women's Health Study of female health professionals.	Mental Health Inventory-5 (MHI-5)	(i) Reduced AF risk in association with greater reported positive effect (ii) Depression not associated with AF risk in multivariable models

Lane et al. [27] (2009)	70 patients with lone AF	To report changes in HRQOL, depression, and anxiety over 12 months. To report if illness perceptions and medication beliefs at time of diagnosis are related to depression and anxiety	(i) Beck Depression Inventory (ii) State and Trait Anxiety (STAI)	(i) Patients with lone AF have low depression rates (ii) Patients with lone AF had higher anxiety scores and the mean anxiety scores did not change over time (iii) Patients who were more concerned about medications had less physical decline over time (iv) Patients who were more anxious had better recovery over time

*Kang [15] (2006) *	*81 patients with AF *	*To report the association between uncertainty, appraisal, symptom severity, and depression *	*Depression (CES-D) *	*Patients with AF had a positive relationship between symptom severity, uncertainty, and depression *

Gehi et al. [28] (2012)	300 patients with documented AF	To report if psychological distress is an important factor in patients report AF symptom severity	(i) Patient Health Questionnaire (PHQ) (ii) Hospital Anxiety and Depression Scale (HADS) (iii) Whitley index (iv) Toronto Atrial Fibrillation Severity Scale	Patients with depression, anxiety, or somatization disorder had more severe AF symptoms regardless of AF burden

**Table 3:** A summary of the articles presented in the section on the connection between the pathophysiology of AF and depression and anxiety. Studies in italics only assessed depression. Studies in bold only assessed anxiety.

Study	Subject size	Aim of the study	Psychological test	Significant findings
The connection between the pathophysiology of AF and depression				

**Eaker et al. [18] (2005)**	**3682 patients from the Framingham offspring study were followed for 10 years**	**To report if tension and anxiety can cause coronary artery disease and AF**	**(i) Framingham tension scale** **(ii) Framingham anxiety scale**	**(i) Tension was a predictor for coronary artery heart disease and mortality.** **(ii) Tension was a predictor for AF.** **(iii) Anxiety was a predictor of total mortality in men and women.** **(iv) Anxiety was a risk factor for incident AF. **

*Rommel et al. [16] (2013)*	*289 patients with AF *	*To report the influence of obesity, physical inactivity, and depression in patients with stable AF *	*9-item Patient Health Questionnaire *	*(i) In unadjusted analyses, mild-to-moderate and severe depressions were associated with increased hs-CRP levels* *(ii) In multivariate analysis, depression was no longer associated with increased hs-CRP levels *

Son and Song [29] (2012)	114 patients with chronic AF	To report if increased hs-CRP levels are associated with depression in an AF population	(i) Type D Scale (ii) Hospital Anxiety and Depression Scale (HADS)	(i) 32% of patients had Type D personality (ii) Type D personality had higher hs-CRP levels

Tully et al. [30] (2011)	226 cardiac surgery patients	To report the incidence of new onset AF	Depression Anxiety Stress Scale (DASS)	(i) 24.8% had postoperative AF (ii) Postoperative anxiety was associated with increased AF in CABG patients

**Table 4:** Summarizes studies presented in “The Benefit of AF Treatment on Depression and Anxiety and Impact of Depression and Anxiety on Treatment Success” section.

	Subjects	Follow-up period	Measure of anxiety, depression	Intervention	Results
Rate and/or rhythm control					

Frasure-Smith et al. [31]	933 patients with AF and CHF	39 ± 18 months	ASI, BDI	Electrical Cardioversion AAD	Higher ASI showed better long-term prognosis with rhythm than rate control (*P* = 0.022)

Lange and Herrmann-Lingen [32]	54 patients with persistent AF	2 months	HADS, DS-14	Electrical cardioversion	(i) An HADS depression score >7 was associated with AF recurrence (85% depressed patients versus 39% nondepressed patients; *P* = 0.004). (ii) HADS anxiety score or type D personality was associated with AF recurrence

Catheter ablation					

Fichtner et al. [33]	133 patients with paroxysmal and persistent AF	4.3 ± 0.5 years	MDI	PVI ± linear or electrogram-guided substrate modification for AF	(i) Regardless of AF type or ablation success, pts. experienced a significant reduction in depressive symptoms (*P* < 0.001) (ii) However, successful ablation led to greater reduction in depressive symptoms than unsuccessful ablation (*P* = 0.039)

Wokhlu et al. [34]	502 patients with paroxysmal, persistent, and longstanding AF	3.1 years	MAFS SF-36	PVI ± linear or electrogram-guided substrate modification for AF	(i) AF ablation produces sustained QoL improvement at 2 years regardless of ablation efficacy (ii) Symptom relief, baseline QoL status, and potential for discontinuing warfarin were found to improve QoL

Sang et al. [35]	166 patients with paroxysmal AF	12 months	SDS, SAS, and SF-36	Catheter ablation AAD	(i) In patients with paroxysmal AF, catheter ablation improves anxiety, depression, and QoL PCS and MCS scores (*P* < 0.001, *P* = 0.001, *P* < 0.001, and *P* = 0.006, resp.) and is superior to AAD therapy in all measures (*P* < 0.001) (ii) Catheter ablation, no AF recurrence, avoidance of warfarin use, higher baseline depression, anxiety scores, and lower baseline QoL scores were associated with reduction in depression and anxiety and improvement in QoL, respectively

Mohanty et al. [36]	660 patients with paroxysmal AF, persistent AF, and long standing persistent AF	12 months	BDI, HAD, STAI, and SF-36	Catheter ablation	(i) Successful ablation was associated with greater reduction in HAD anxiety, HAD depression, and BDI and greater improvement in SF-36 PCS scores (*P* = 0.003; *P* < 0.001; *P* = 0.024; *P* < 0.001, resp.) (ii) STAI scores did not show any association with ablation success

Yu et al. [37]	146 patients with persistent AF	12 months	SAS, SDS	CVPA AAD	(i) Anxiety and depression improved after successful ablation (*P* < 0.01 for both) (ii) There was no change in anxiety or depression scores in the AAD from baseline to 12 months after enrollment (iii) SAS and SDS scores ≥ were independent risk factors of AF recurrence one-year status after CPVA (*P* = 0.02 for both)

Complementary and alternative medicine					

Lakkireddy et al. [38]	49 patients with paroxysmal AF	3-month control (patients are own control) 3-month yoga therapy	SAS, SDS, and SF-36	Iyengar yoga instruction for 60 min at least 2 times weekly	(i) In patients with paroxysmal AF, yoga reduced the number of symptomatic AF episodes, symptomatic non-AF episodes, and asymptomatic AF episodes (*P* < 0.001 for all). (ii) Yoga improved anxiety and depression scores (*P* < 0.001) and QoL parameters of physical functioning, general health, vitality, social functioning, and mental health (*P* = 0.017, *P* < 0.001, *P* < 0.001, *P* = 0.019, and *P* < 0.001)

SAS: Zung Self-Rating Anxiety Scale; SDS: depression symptoms Zung Self-Rating Depression Scale; SF-36: Medical Outcomes Short Form-36; BDI: Becks Depression Inventory; HAD: Hospital Anxiety and Depression scale; MDI: major depression inventory; MAFSI: Mayo AF-Specific Symptom Inventory; STAI: State Trait Anxiety Inventory; Qol: quality of life.
